# Quaternary structure is an essential component that contributes to the sophisticated allosteric regulation mechanism in a key enzyme from *Mycobacterium tuberculosis*

**DOI:** 10.1371/journal.pone.0180052

**Published:** 2017-06-30

**Authors:** Wanting Jiao, Nicola J. Blackmore, Ali Reza Nazmi, Emily J. Parker

**Affiliations:** 1 Maurice Wilkins Centre for Molecular Biodiscovery, Biomolecular Interaction Centre and Department of Chemistry, University of Canterbury, Christchurch, New Zealand; 2 Ferrier Research Institute, Victoria University of Wellington, Wellington, New Zealand; Griffith University, AUSTRALIA

## Abstract

The first enzyme of the shikimate pathway, 3-deoxy-D-*arabino*-heptulosonate 7-phosphate synthase (DAH7PS), adopts a range of distinct allosteric regulation mechanisms in different organisms, related to different quaternary assemblies. DAH7PS from *Mycobacterium tuberculosis* (*Mtu*DAH7PS) is a homotetramer, with the allosteric sites in close proximity to the interfaces. Here we examine the importance of the quaternary structure on catalysis and regulation, by amino acid substitution targeting the tetramer interface of *Mtu*DAH7PS. Using only single amino acid substitutions either in, or remote from the interface, two dimeric variants of *Mtu*DAH7PS (*Mtu*DAH7PS_F227D_ and *Mtu*DAH7PS_G232P_) were successfully generated. Both dimeric variants maintained activity due to the distance between the sites of amino acid substitution and the active sites, but attenuated catalytic efficiency was observed. Both dimeric variants showed significantly disrupted allosteric regulation with greatly impaired binding affinity for one of the allosteric ligands. Molecular dynamics simulations revealed changes in protein dynamics and average conformations in the dimeric variant caused by amino acid substitution remote to the tetramer interface (*Mtu*DAH7PS_G232P_), which are consistent with the observed reduction in catalytic efficiency and loss of allosteric response.

## Introduction

Protein complexes with different quaternary structures are essential functional modules within protein interaction networks in the cellular environment [1]. Quaternary structure may be involved in protein function and allosteric regulation, and this has been demonstrated in various protein families such as receptor heteromers, cGMP-dependent protein kinases, transport proteins, and the human ribonuclease H2 complex [2–5]. Here, we have investigated the importance of the quaternary structure for the function and regulation of the biosynthetic enzyme 3-deoxy-d-*arabino*-heptulosonate 7-phosphate synthase (DAH7PS).

DAH7PS catalyzes an aldol condensation reaction between phosphoenolpyruvate (PEP) and erythrose 4-phosphate (E4P) to produce 3-deoxy-d-*arabino*-heptulosonate 7-phosphate (DAH7P), which is the first committed step of the shikimate pathway. The shikimate pathway operates in microorganisms and plants and is responsible for the de novo biosynthesis of aromatic amino acids [6]. As the first enzyme of a critical biosynthetic pathway, DAH7PS is a major flux control point for the shikimate pathway. DAH7PS can be classified into two types [7]. Type I enzymes are smaller with molecular masses less than 40 kDa, and many have been well characterized [8–13]. Type II enzymes are larger with molecular masses over 50 kDa. The only member of type II DAH7PS that has been structurally characterized is the DAH7PS from *Mycobacterium tuberculosis* (*Mtu*DAH7PS) [14]. Previous studies have shown that *Mtu*DAH7PS, unlike the DAH7PS enzymes characterized so far from other organisms, adopts a highly sophisticated mechanism of allosteric control [15–18]. Whilst remaining insensitive to individual aromatic amino acids, binary combinations that involve Trp (Trp+Phe and Trp+Tyr) result in a significant loss of enzyme activity, and the ternary combination of all three aromatic amino acids completely abolishes the reaction, revealing synergy in the response to different allosteric effectors [18, 19]. Moreover, *Mtu*DAH7PS forms a complex and shares its allostery with *M*. *tuberculosis* chorismate mutase (*Mtu*CM), which acts at the branch point that connects the shikimate pathway to Phe and Tyr production [20–22].

**Fig 1:**
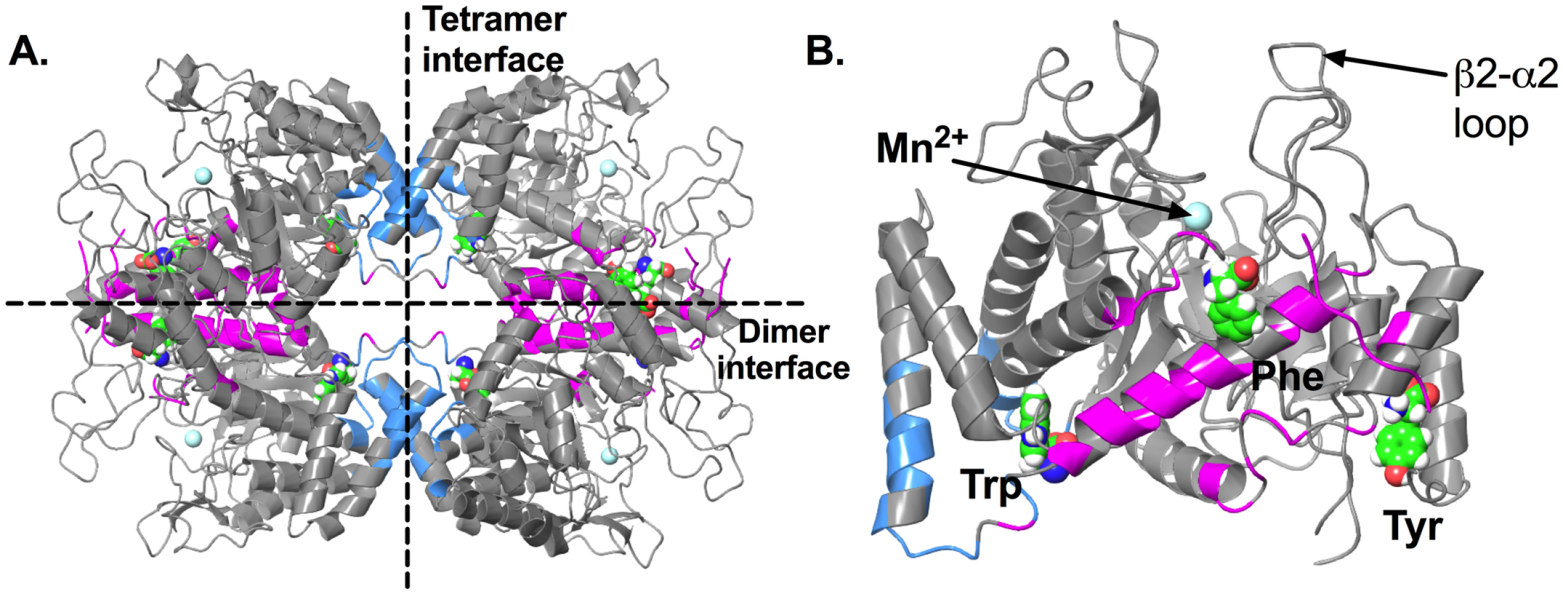
Crystal structure of *Mtu*DAH7PS. (A) Tetramer of *Mtu*DAH7PS (PDB 5CKV), dimer and tetramer interfaces are indicated with dashed lines. Residues contributing to the dimer and tetramer interfaces are colored pink and blue respectively. (B) Monomeric unit of *Mtu*DAH7PS in complex with allosteric ligands Phe, Trp and Tyr (PDB 5CKV), the three aromatic amino acids are displayed as spheres with green carbon atoms, the active site metal ion Mn^2+^ is shown as cyan sphere.

Crystal structures of ligand-free *Mtu*DAH7PS revealed a homotetramer quaternary structure assembled from (β/α)_8_ TIM barrel subunits [14]. Each of the core barrels hosts an active site and is decorated by accessory structural elements, including a two-stranded β-sheets (β0), a three helix extension at the N-terminus (α0a, α0b and α0c) and two inserted helices (α2a and α2b) [18]. These accessory structural elements contribute to the formation of the dimer and tetramer interfaces (Fig 1A). Previous studies have identified active site residues of *Mtu*DAH7PS that are responsible for the binding of substrates PEP and E4P through crystallography [11, 12, 23]. Residues Arg126, Lys306, Arg337, Arg284 and Glu283 stabilize the binding of PEP. Residues from the β2-α2 loop of the active site, including Arg135 and Ser136, play key roles in the positioning of the E4P phosphate. The dynamics of residues in the β2-α2 loop have been shown to strongly affect the enzyme’s response to E4P [24].

Crystal structures of *Mtu*DAH7PS in complex with different ligands showed that there are three distinct allosteric binding sites [19, 21, 22]. Two of these are located directly at the dimer and tetramer interfaces, and bind Phe and Trp respectively. The third binding site, which is selective for Tyr, is located on the exterior surface of the enzyme near the dimer interface (Fig 1B) [18, 19].

*Mtu*CM binds to *Mtu*DAH7PS across the tetramer interface to form a hetero-octameric complex, which results in the substantial activation of chorismate mutase activity [20]. Binding of Phe and/or Tyr to their allosteric sites at or near the dimer interface of *Mtu*DAH7PS has been shown to destabilize the *Mtu*CM interaction, leading to dissociation of the hetero-octamer, and a reduction of *Mtu*CM activity [21, 22]. The intricate network of interconnecting binding sites displayed by *Mtu*DAH7PS creates a highly sophisticated allosteric system controlling both entry into the pathway for aromatic amino acid biosynthesis and branch point partitioning. It should be noted that while DAH7PS enzymes from other sources share similar catalytic barrels, this combination of quaternary structure and allosteric binding sites appears to be unique to *Mtu*DAH7PS [18, 19, 25–27]. Due to a lack of structural information about other type II DAH7PS, this as yet unique quaternary structure may yet prove to be a feature of all type II DAH7PS enzymes.

The observation that *Mtu*DAH7PS interfaces are closely associated with the allosteric sites implies the significance of the quaternary structure for delivery of the allostery and possibly function. Here, we investigate the role of quaternary structure in the function and regulation of *Mtu*DAH7PS using dimeric variants of *Mtu*DAH7PS generated by amino acid substitution targeting the tetramer interface. Our results demonstrate that changing the quaternary structure of *Mtu*DAH7PS not only significantly reduces its allosteric response to aromatic amino acids, but also impairs catalytic function, exposing the dynamic connections between remote sites.

## Results

### Identification of target residues for mutagenesis

While the asymmetric unit in the X-ray crystal structures of *Mtu*DAH7PS is a dimer, the biological assembly of this enzyme is tetrameric. In addition, solution phase experiments such as small angle X-ray scattering (SAXS) experiments have confirmed that the tetramer is observed in solution [24]. In the ligand-free *Mtu*DAH7PS crystal structure (PDB 3NV8), the dimer interface involves more than 50 residues from each chain and has a buried area of 1860 Å^2^. The tetramer interface, in contrast, is less extensive with a buried area of 990 Å^2^ and the involvement of only 24 residues from each chain [28, 29].

**Fig 2:**
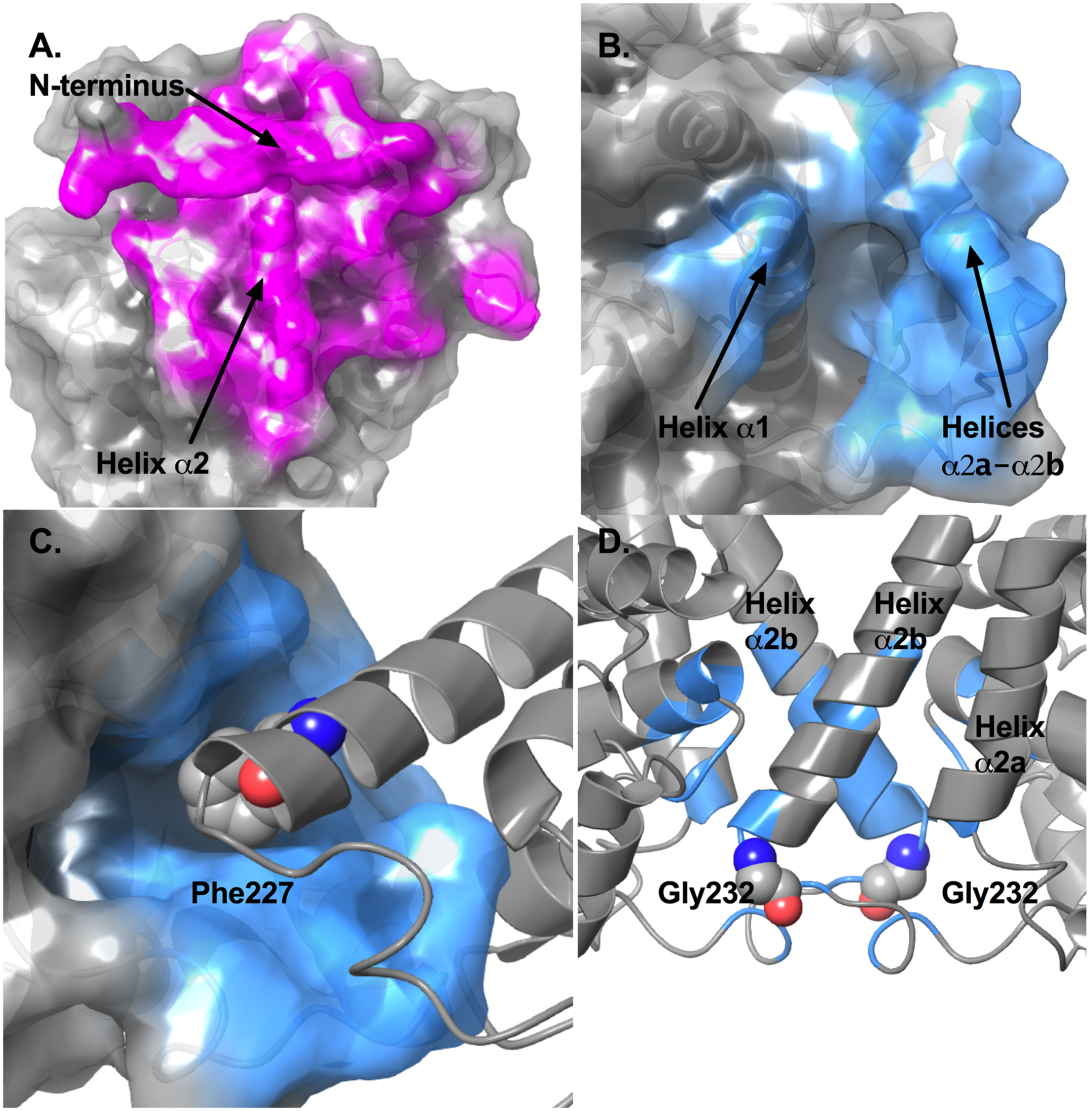
Analysis of the dimer and tetramer interfaces in *Mtu*DAH7PS. (A) Surface representation of the dimer interface of *Mtu*DAH7PS (PDB 3NV8). Residues contributing to this interface are displayed as red sticks. Residues identified to contribute to the dimer interface by PISA include residues 2–13, 15, 44, 47–48, 51, 54–58, 60, 62–63, 92, 94–96, 99, 165–167, 170–171, 173–175, 177–178, 180–182, 184–186, 189, 260, 263 and 265 of chain A, and residues 1, 3–13, 47–48, 51, 54–58, 60, 62–63, 91–92, 94–97, 99–100, 165, 167–168, 170–171, 173–175, 177–182, 184–186, 188–190,296, and 260 of chain B. (B) Surface representation of the tetramer interface of *Mtu*DAH7PS (PDB 3NV8). Residues contributing to this interface are displayed as blue sticks. Residue numbers are described in the main text. (C) Hydrophobic grove in the tetramer interface and the interaction with Phe227 from the other chain. (D) Positions of the two Gly232 residues on the tetramer interface, succeeding helices α2a and α2b.

The larger dimer interface is formed between residues from the N-termini, part of helix α0b and the α0b-α0c loop, part of the β1-α1 loop, and helix α2 (Fig 2A). Backbone hydrogen bonds can be found between the N-terminal residues to stabilize the anti-parallel β-sheet that caps the allosteric binding sites for Phe. Other polar interactions contributing to the dimer interface include a hydrogen bond between the side chains of Asn181 of chain A and Arg184 of chain B, and hydrogen bonds between Asn181 and the backbone carbonyl groups of Pro57 and Val58 from the other chain. Salt bridge interactions are formed between Asp10 of chain A and Arg171 of chain B.

The tetramer interface consists of residues mostly from the inserted helices α2a to α2b (residues 194, 220, 221, 223–233, 237, 238) and part of the α1 helix (residues 111, 114, 115, 117–120) (Fig 2B). The tetramer interface is largely hydrophobic, with only 6 hydrogen bonds identified when analyzed using PISA [28, 29]. These include the two equivalent hydrogen bonds formed between the side chain of Arg223 and the backbone carbonyl of Arg461, the two equivalent hydrogen bonds formed between side chains of Arg226 and Ser118, and the two equivalent hydrogen bonds formed between the side chain of Tyr115 and backbone carbonyl of Glu220. Inspection of the tetramer interface also reveals a small hydrophobic groove. Residues contributing to this hydrophobic groove include those from the α1 helix (Val111 and Tyr115), the α2-α2a loop (Leu194) and the inserted helices α2a (His198) and α2b (Ile221, Leu225 and Met228). Phe227 located on the α2b helix of the subunit across the tetramer interface protrudes into this groove (Fig 2C).

In order to investigate the influence of quaternary structure on catalytic function and allosteric response, variant enzymes of *Mtu*DAH7PS were proposed to favor dimeric over tetrameric states of the enzyme. Two approaches to disrupt the tetramer interface were explored. The first was to disrupt physical interactions formed at the tetramer interface; for this purpose Phe227 was substituted by Asp (variant *Mtu*DAH7PS_F227D_) to disturb the hydrophobic interactions formed at this position, and weaken the tetramer interface. The second approach taken was to disrupt the dynamic properties of regions involved in forming the tetramer interface, based on the observations from previous studies that molecular dynamics play a critical role in the allosteric regulation and communication networks of *Mtu*DAH7PS [24]. Therefore, Gly232 located on the loop succeeding the two inserted helices (α2a and α2b) that contribute to the tetramer interface, was identified as a potential target to effect dynamic disruption to the tetramer (Fig 2D). Hence, Gly232 was substituted by Pro (creating *Mtu*DAH7PS_G232P_). Both variant enzymes were expressed and purified using standard procedures developed for the wild-type protein [14, 18, 21].

### Tetramer formation is disrupted in variant enzymes of *Mtu*DAH7PS

**Fig 3:**
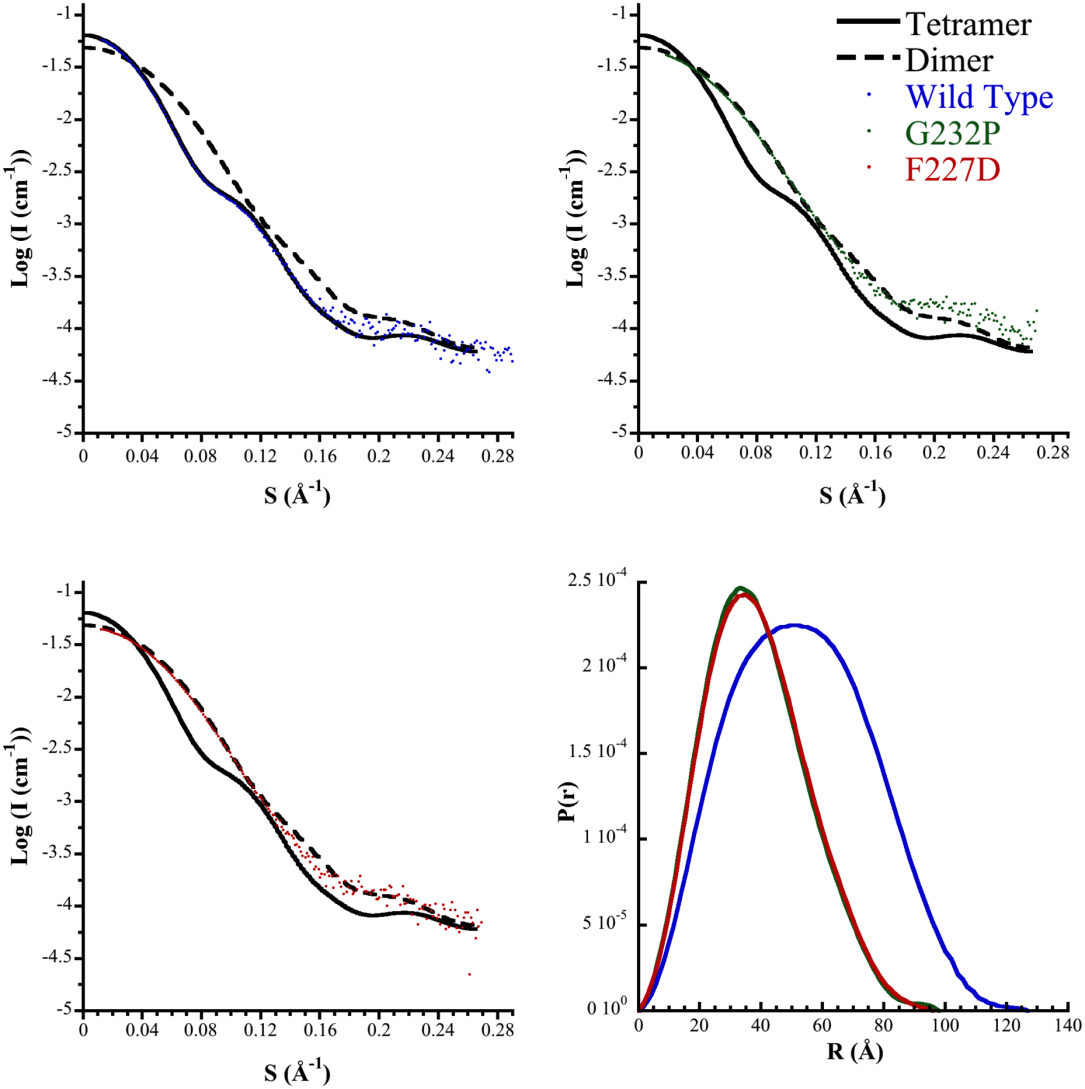
SAXS profiles of *Mtu*DAH7PS_WT_ (blue), *Mtu*DAH7PS_G232P_ (green), and *Mtu*DAH7PS_F227D_ (red). Also shown is the theoretical scattering for the tetrameric (full biological assembly, black) and dimer (asymmetric unit, black dash) of *Mtu*DAH7PS from PDB code 3NV8, and the corresponding pair-wise distribution profiles. The lowest χ values calculated by CRYSOL were determined to be 0.986 and 2.36 for *Mtu*DAH7PS_G232P_ and *Mtu*DAH7PS_F227D_ respectively.

The quaternary structures of the variant enzymes in solution were analyzed by small angle X-ray scattering (SAXS). The scattering profiles of *Mtu*DAH7PS_G232P_ and *Mtu*DAH7PS_F227D_ were found to be very similar to each other, but quite distinct from the that of the WT enzyme and the profile predicted from the *Mtu*DAH7PS tetrameric crystal structure (Fig 3). In contrast, the profile predicted for a dimer generated by disrupting the tetramer interface, provided a better fit to the data. Consistent with a change in quaternary structure in the two variants, *D*_max_ values (a measure of the maximum dimension of the scattering species) of 98 Å and 96 Å were observed for *Mtu*DAH7PS_G232P_ and *Mtu*DAH7PS_F227D_ respectively (in comparison with *D*_max_ of 125 Å for the wild type [24]).

**Fig 4:**
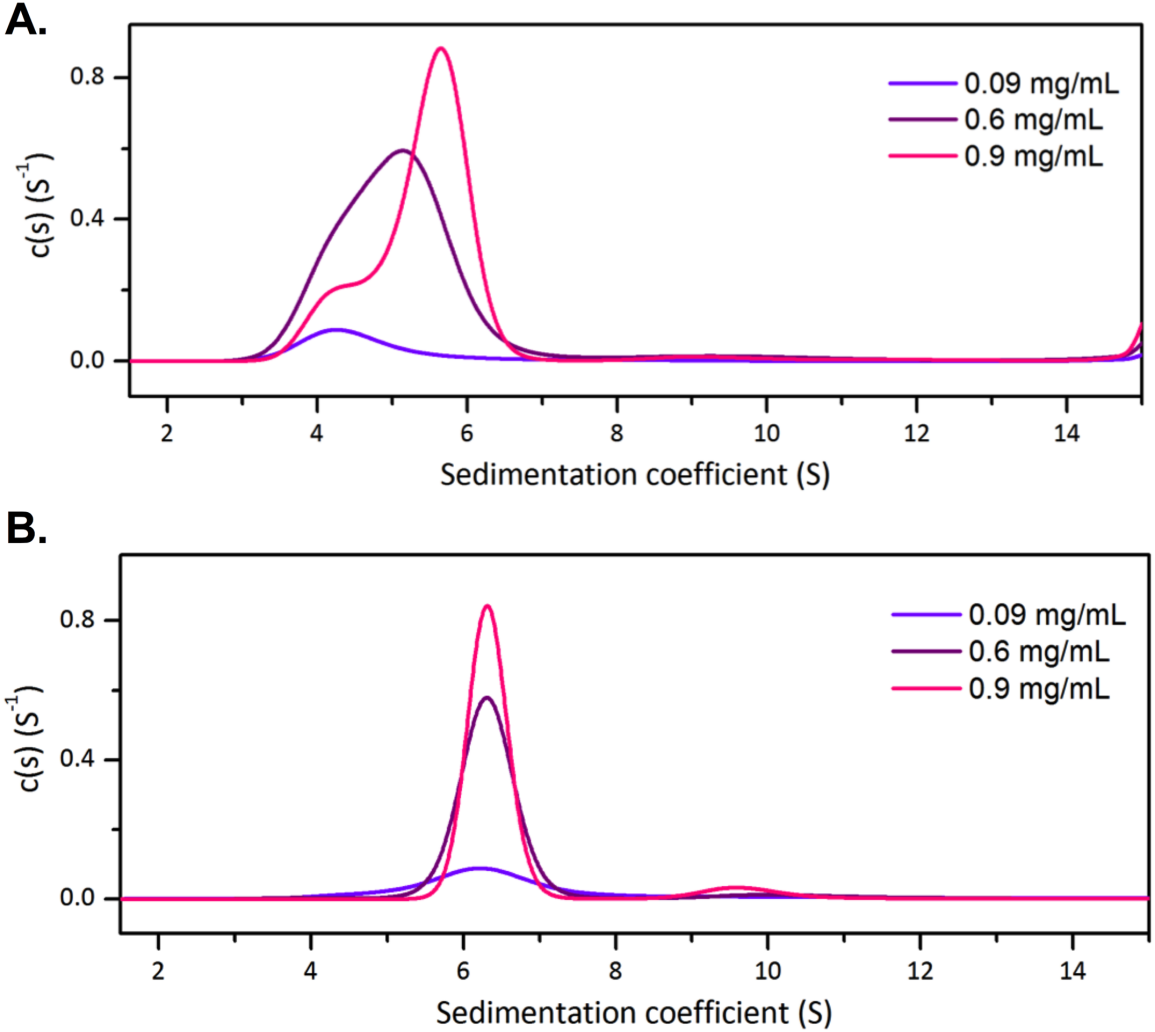
Normalized sedimentation coefficient distribution functions from AUC data for *Mtu*DAH7PS_G232P_. AUC data were collected in the (A) absence and (B) presence of 100 μM Trp and 100 μM Phe. *Mtu*DAH7PS_G232P_ was analyzed at 0.09 mg/mL (violet), 0.6 mg/mL (purple) and 0.9 mg/mL (pink).

To further analyze the changes in quaternary structures at low protein concentrations, analytical ultracentrifugation (AUC) was used to further investigate the quaternary structure of one of the dimeric variants, *Mtu*DAH7PS_G232P_, in solution. Sedimentation velocity experiments were carried out at three protein concentrations under the conditions previously described for the wild type enzyme [21]. At the lowest analyzed concentration, 0.09 mg/mL, a major species with a sedimentation coefficient of 4.0 S can be observed (Fig 4A). The calculated molecular mass for this species is 54 kDa, which is in good agreement with monomeric mass of *Mtu*DAH7PS (~51 kDa). At a protein concentration of 0.6 mg/mL, a broadened peak with sedimentation coefficient of 5.6 S is observed indicating the presence of a mixture of monomeric and dimeric species. At 0.9 mg/mL, two species are observed. The major species has a sedimentation coefficient of 5.7 S and a minor species has a sedimentation coefficient of 4.1 S (which corresponded to the monomeric species). The increase in sedimentation coefficient as protein concentration increases suggests the presence of a concentration dependent equilibrium between monomeric and dimeric species of *Mtu*DAH7PS_G232P_.

When samples of *Mtu*DAH7PS_G232P_ were spiked with 100 μM each of Trp and Phe, analysis showed only one major species with sedimentation coefficient of 6.1 S for all three tested protein concentrations (Fig 4B). This species has a calculated molecular mass of ~92 kDa in close agreement with the molecular mass of dimeric *Mtu*DAH7PS (~102 kDa). Hence, the addition of Trp and Phe had shifted the equilibrium towards the dimeric form of *Mtu*DAH7PS_G232P_. As Phe binds at the dimer interface, Phe was most likely to be primarily responsible for the stabilization of the *Mtu*DAH7PS_G232P_ dimer by strengthening the interactions between the two monomers.

### Dimeric *Mtu*DAH7PS variants are catalytically active, but poorly inhibited by aromatic amino acids

Both dimeric *Mtu*DAH7PS_G232P_ and *Mtu*DAH7PS_F227D_ variants were found to be catalytically active (Table 1). For both enzymes, a significant change of the E4P Michaelis constant (*K*_m_^E4P^) was noted, with increases of 11-fold and 20-fold relative to the wild-type enzyme for *Mtu*DAH7PS_G232P_ and *Mtu*DAH7PS_F227D_ respectively. The *K*_m_ values for PEP for both variants also showed large increases of approximately 5-6-fold relative to the wild-type enzyme. Previous studies have shown that upon binding of allosteric regulators (100 μM Phe and 100 μM Trp) to wild-type enzyme, the reaction rate response with respect to E4P concentration changed from typical hyperbolic Michaelis-Menten curve to a sigmoidal response indicating homotropic cooperativity with respect to E4P, and a shift of apparent *K*_m_^E4P^ from 37 μM to a E4P_0.5_ of 382 μM was observed [18]. In this study, the signature homotropic cooperativity with respect to E4P is not observed in either dimeric variants. Although the large increase in measured *K*_m_ values for PEP and E4P in the dimeric variants suggests that dimerization may partially mimic the effect of allosteric ligand binding, the lack of homotropic cooperativity response with respect to E4P indicates that dimerization does not result in the same effect as that observed in the allosterically inhibited state of the wild-type enzyme. The turnover number (*k*_cat_) determined for both *Mtu*DAH7PS variants was elevated, however, the relatively large changes to the *K*_m_ values mean that the efficiency of both dimeric variants is considerably impaired.

**Table 1:** Kinetic parameters of wild-type *Mtu*DAH7PS, *Mtu*DAH7PS_G232P_ and *Mtu*DAH7PS_F227D_ determined from ternary complex rate equation.

*Mtu*DAH7PS	*K*_m_^E4P^ (μM)	*K*_m_^PEP^ (μM)	*k*_cat_ (s^-1^)
Wild-type	29 ± 5	15 ± 4	6.7 ± 0.4
G232P	330 ± 10	90 ± 20	15 ± 7
F227D	590 ± 60	80 ± 20	8 ± 3

**Fig 5:**
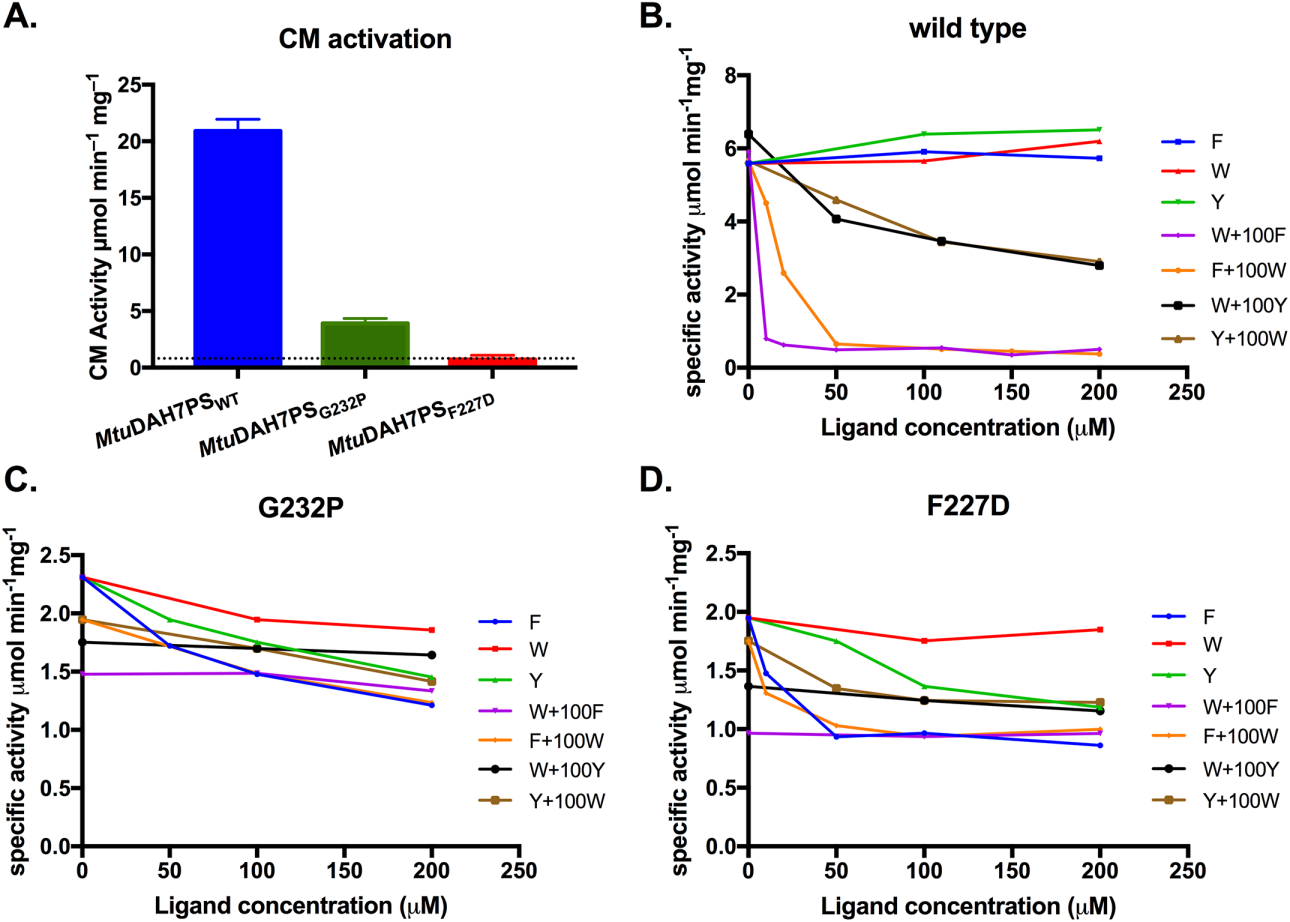
Activities of *Mtu*CM, wild type and variant *Mtu*DAH7PS. (A) Activation of *Mtu*CM activity in the presence of ten-fold molar excess of wild-type *Mtu*DAH7PS, *Mtu*DAH7PS_G232P_ or *Mtu*DAH7PS_F227D_. The dashed line indicates the activity of *Mtu*CM alone. Assays conducted with 150 μM chorismic acid and 60 nM *Mtu*CM except for *Mtu*CM alone where 90 nM was used. Specific activity of (B) wild-type *Mtu*DAH7PS and (C) *Mtu*DAH7PS_G232P_ and (D) *Mtu*DAH7PS_F227D_ in the presence of various single and binary combinations of aromatic amino acids. For binary combinations, the background concentration of the indicated ligand was held at 100 μM. Assays conducted in the presence of 150 μM PEP and 150 μM E4P.

As expected from the known site of interaction of *Mtu*CM [20], disruption of the tetramer interface of *Mtu*DAH7PS significantly reduced its ability to activate *Mtu*CM in comparison to tetrameric wild-type (Fig 5A).

In order to investigate the impact of the quaternary structure change on the allosteric regulation of *Mtu*DAH7PS, inhibition studies were conducted on both variant enzymes (Fig 5B–5D). Due to dimerization of E4P at high concentrations, the inhibition assay was conducted with a standard saturating E4P concentration for the wild-type enzyme (150 μM); this same concentration of E4P for the dimeric variants was not saturating, given the large increase in *K*_m_ values. DAH7PS activity was determined in the presence of both single and binary combinations of the aromatic amino acids. Combinations of amino acids including Trp and Phe, which caused 90% inhibition in the wild-type enzyme (95% inhibition with unsaturated E4P concentration of 50 μM [18]), did not have a substantial effect on activity of the two dimeric variants (~50% inhibition, but only at relatively high ligand concentrations, Fig 5B–5D). Combinations involving Trp and Tyr had even smaller inhibitory effects, showing only 30%-40% inhibition in comparison with the 50% inhibition (84% inhibition with 50 μM E4P [18]) observed for the wild-type enzyme. For both dimeric variants, some inhibition by Phe (50% inhibition), and to a lesser extent by Tyr (40% inhibition), was observed. Although no inhibition was observed for wild-type enzyme by Phe or Tyr alone under saturating E4P concentrations, small inhibitory effect can be observed (50% inhibition with 200 μM of Phe and 47% inhibition with 200 μM of Tyr) at unsaturated E4P concentration of 50 μM, caused by small shifts in E4P *K*_m_ values in the presence of single aromatic amino acids [18].

### *Mtu*DAH7PS_G232P_ and *Mtu*DAH7PS_F227D_ bind Trp and Phe

Isothermal titration calorimetry (ITC) was performed to assess the binding of Trp and Phe to the dimeric variant enzymes. Despite the attenuated inhibitory response to the amino acids, binding was observed (Table 2). The dissociation constants for Phe binding, known to bind at the preserved dimeric interface, were very similar to that for the wild-type enzyme (*K*_d_ = 21 ± 1 μM) [24]. On the other hand, Trp binding was significantly attenuated in comparison with wild-type enzyme (*K*_d_ = 4.7 ± 0.1 μM) [24], consistent with the proximity of the Trp binding site to the disrupted interface. Interestingly, the binding of Trp was not improved when either dimeric variant was pre-incubated with Phe (in contrast to a 4-fold reduction in *K*_d_ of Trp in wild-type enzyme after pre-incubated with Phe [24]), suggesting that the communication between Phe and Trp binding sites that was observed in the wild type enzyme had been disrupted by the loss of the tetrameric quaternary structure.

**Table 2:** Dissociation constants (*K*_d_) of Phe and Trp binding to *Mtu*DAH7PS_G232P_ and *Mtu*DAH7PS_F227D_ determined from ITC.

Titrated ligand	Background ligand	*Mtu*DAH7PS_G232P_ *K*_d_ (μM)	*Mtu*DAH7PS_F227D_ *K*_d_ (μM)
Phe		17 ± 2 ^a^	15.9 ± 0.9 ^c^
Trp		150 ± 10 ^b^	200 ± 20 ^d^
Trp	Phe	210± 30 ^e^	220± 20 ^f^

^a^28 μM of *Mtu*DAH7PS_G232P_ with 900 μM Phe titrant.

^b^30 μM *Mtu*DAH7PS_G232P_ with 3 mM Trp titrant.

^c^32 μM of *Mtu*DAH7PS_F227D_ with 600 μM Phe titrant.

^d^32 μM *Mtu*DAH7PS_F227D_ with 5 mM Trp titrant.

^e^24 μM *Mtu*DAH7PS_G232P_ and a background of 50 μM Phe present in the cell with a 5 mM Trp titrant.

^f^28 μM *Mtu*DAH7PS_F227D_ and a background of 50 μM Phe present in the cell with a 5 mM Trp titrant.

### Dimerization is accompanied by changes in protein dynamics

The cause of dimerization in *Mtu*DAH7PS_F227D_ variant is quite clear as the amino acid substitution introduced directly breaks contacts involved in the formation of the tetramer interface. However, the reasons for dimerization, and the effects of this change in quaternary structure were not immediately obvious for the *Mtu*DAH7PS_G232P_ variant. Given that protein dynamics has previously been shown to play an important role in the allosteric regulation of *Mtu*DAH7PS, molecular dynamics (MD) simulations were conducted for the dimeric variant *Mtu*DAH7PS_G232P_, the theoretical wild-type dimer *Mtu*DAH7PS_WT_^dimer^ and the tetrameric *Mtu*DAH7PS_WT_ in order to examine the effects of dimerization on the dynamic properties of this enzyme. As no experimental structures are available, both dimeric species were generated *in silico* based on the crystal structure of wild-type protein *Mtu*DAH7PS (PDB 3NV8). The MD simulations were conducted for 464.1 ns, 429.9 ns and 540.9 ns for *Mtu*DAH7PS_G232P_, *Mtu*DAH7PS_WT_ and *Mtu*DAH7PS_WT_^dimer^ systems respectively. All systems were equilibrated and analysis were conducted using only trajectories from the equilibrated time period (S1 Fig).

**Fig 6:**
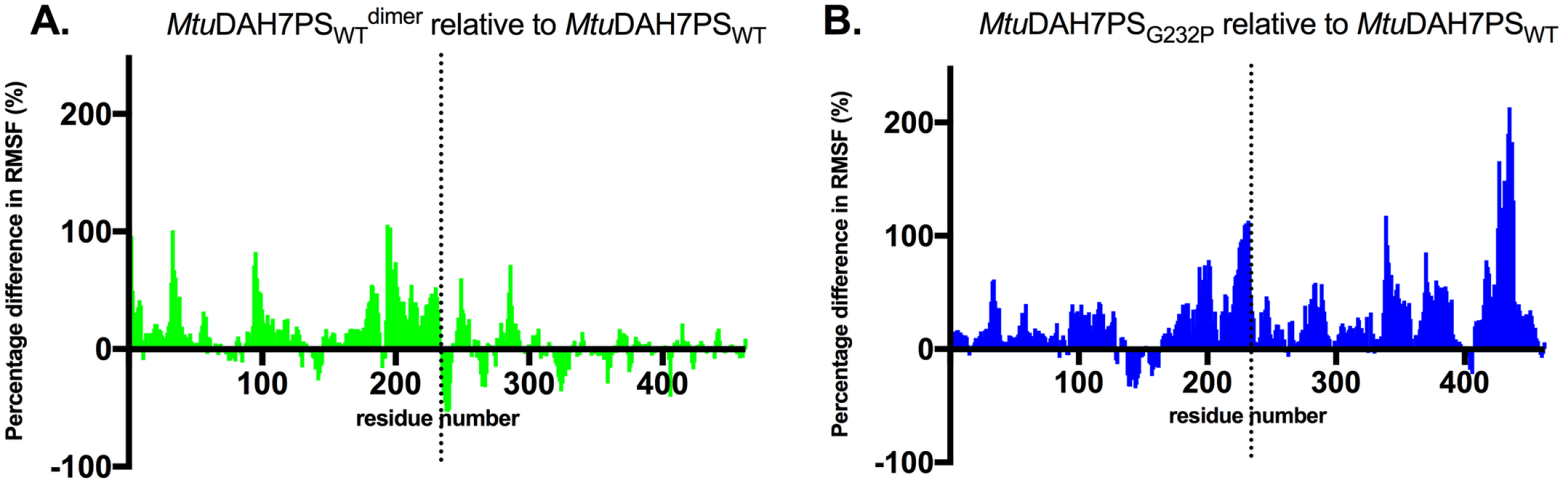
Percentage difference in residue RMSF values. Percentage difference in residue RMSF values between (A) tetrameric *Mtu*DAH7PS_WT_ and dimeric variant *Mtu*DAH7PS_G232P_ and (B) tetrameric *Mtu*DAH7PS_WT_ and theoretical dimer *Mtu*DAH7PS_wt_^dimer^. The dashed line indicates the position of the substitution G232P along the amino acid sequence of *Mtu*DAH7PS. Residue RMSF values were measured in reference to the corresponding MD average conformation using trajectories obtained from equilibrated time period in the MD simulation for each system (50–464.1 ns, 70–429.9 ns and 120–540.9 ns for *Mtu*DAH7PS_G232P_, *Mtu*DAH7PS_WT_ and theoretical dimer *Mtu*DAH7PS_wt_^dimer^, respectively). Chain-average RMSF values were calculated and compared. Percentage difference is calculated by (*RMSF*_*dimer*_ − *RMSF*_*tetramer*_) ÷ *RMSF*_*tetramer*_ × 100.

RMSF values were calculated for all three systems. Due to the presence of multiple chains, the chain-averaged RMSF values were calculated for comparison. A comparison between the calculated average RMSF values for *Mtu*DAH7PS_WT_ and temperature factors obtained from ligand-free crystal structure of *Mtu*DAH7PS_WT_ (PDB 3NV8) showed good correlation in predicted flexible regions (S2 Fig). For both dimeric systems (*Mtu*DAH7PS_WT_^dimer^ and *Mtu*DAH7PS_G232P_), RMSF values were measured using the *α*-carbons for each individual residue and compared to those measured for the tetrameric wild-type protein (Fig 6, and calculated RMSF values for *Mtu*DAH7PS_WT_ is provided in Figure A in S2 Fig). The change in flexibility is then reflected by calculating the differences in average RMSF values. Due to the loss of restraint from the tetramer interface, both dimeric systems appeared to be more flexible in general compared to the tetrameric wild type. Interestingly, residues 300–462 did not show large increases in flexibility in the theoretical dimer compared to those calculated for *Mtu*DAH7PS_G232P_, which suggests that whereas the general increase in flexibility in *Mtu*DAH7PS_G232P_ is likely to be contributed by the loss of the tetramer interface, the increase in flexibility for residues 300–462 appears to arise from the G232P substitution.

**Fig 7:**
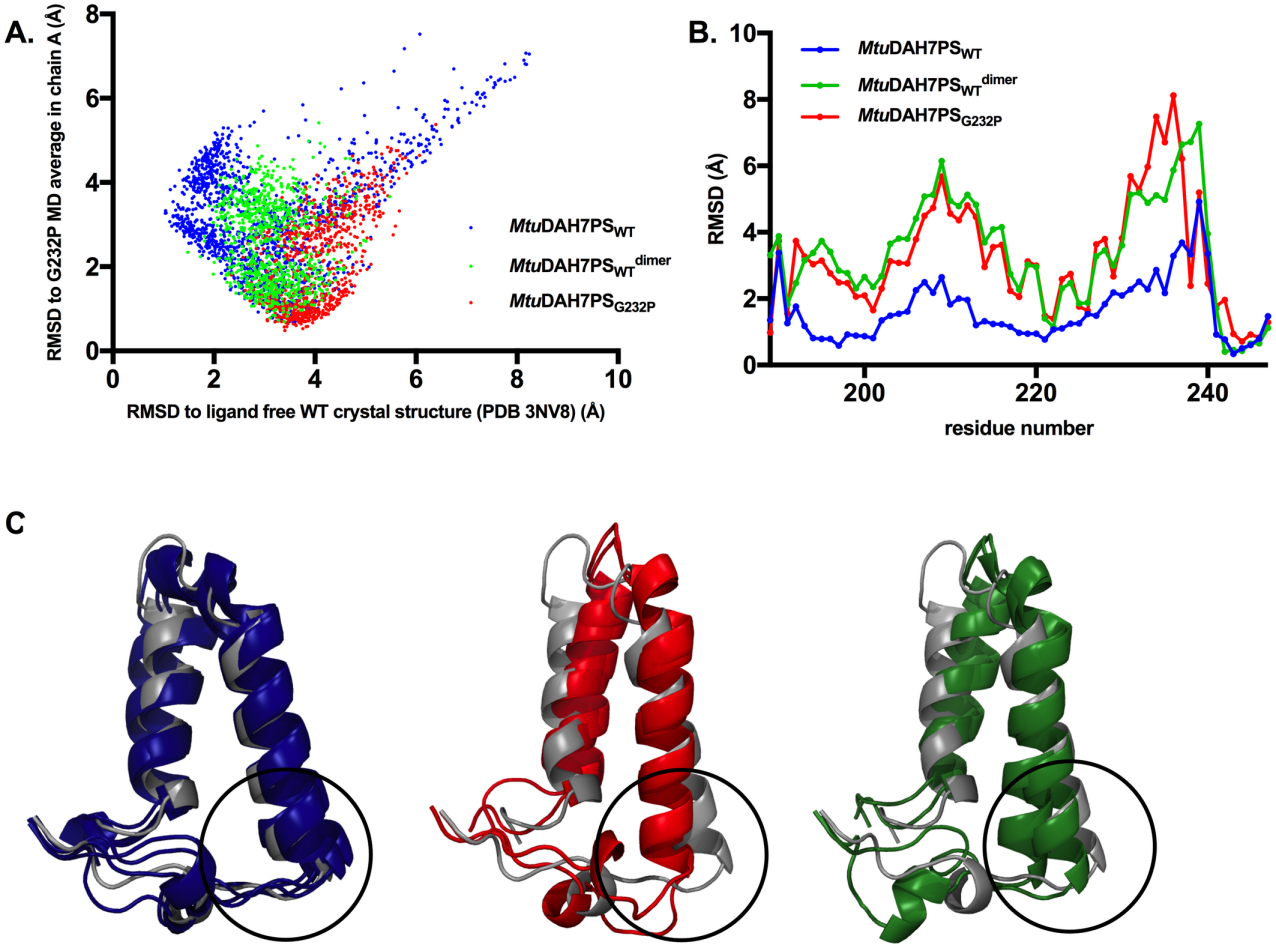
Analysis of conformational ensembles from MD simulations. (A) MD conformational ensemble sampled by loop 2 (residues 130 to 153) during equilibrated time period of MD simulations for tetrameric *Mtu*DAH7PS_WT_ (blue), dimeric variant *Mtu*DAH7PS_G232P_ (red) and the theoretical dimer *Mtu*DAH7PS_WT_^dimer^ (green). RMSD values of Cα atoms on loop 2 residues (130 to 153) were measured in reference to both the MD average of *Mtu*DAH7PS_G232P_ variant and crystal structure of ligand free *Mtu*DAH7PS_WT_. (B) Chain-averaged α-carbon RMSD values of MD average conformations of *Mtu*DAH7PS_WT_, *Mtu*DAH7PS_wt_^dimer^, and *Mtu*DAH7PS_G232P_ in comparison with crystal structure of ligand free *Mtu*DAH7PS (PDB 3NV8), for residue range 189–247. (C) Superimposition between ligand free crystal structure of *Mtu*DAH7PS (PDB 3NV8, grey) and MD average conformations of each chain from MD simulations of *Mtu*DH7PS_WT_ (blue), *Mtu*DAH7PS_G232P_ (red) and *Mtu*DAH7PS_WT_^dimer^ (green), only residues 190–243 are displayed for clarity. Regions that are responsible for forming the tetramer interface are highlighted in black circles.

Residues 130 to 164, which comprise the β2-α2 loop in the active site, showed reduced flexibility in the dimeric systems. This loop is responsible for the binding of E4P and has been shown to bind to the E4P-mimicking portion of an *Mtu*DAH7PS inhibitor designed to resemble the reaction intermediate [23]. Further examination of the backbone conformational ensembles sampled by the β2-α2 loop (residues 130 to 153) was conducted by measuring the *α*-carbon RMSD values in comparison to β2-α2 loop conformations in both the ligand free crystal structure of *Mtu*DAH7PS_WT_ (PDB 3NV8) and the MD average conformation of chain A in *Mtu*DAH7PS_G232P_ (Fig 7A). The results showed that the β2-α2 loop in *Mtu*DAH7PS_WT_ samples a large range of conformations; only upon dimerization in the theoretical dimer *Mtu*DAH7PS_WT_^dimer^, a decrease in the number of accessible conformations is observed; whereas in the dimeric variant *Mtu*DAH7PS_G232P_ the effect is more pronounced with an even smaller subset of conformations accessible. The decreasing number of accessible conformations for the β2-α2 loop in the dimeric species may mean that the catalytically relevant conformations of loop 2 for E4P binding and reaction with PEP (as represented by the conformations with low RMSD values to ligand free *Mtu*DAH7PS_WT_ crystal structure) are not being sampled, which may contribute to the observed impairment of the catalytic efficiency in the dimeric species.

### Dimerization is accompanied by changes in average conformations around regions involved in tetramer interface formation

Out of the two mutated residues that disrupted quaternary structures (G232 and F227), F227 is directly involved in physical interactions within the tetramer interface, and the amino acid substitution impairs dimerization directly. However, it is not apparent from an inspection of the crystal structure of ligand free *Mtu*DAH7PS_WT_ how the substitution of Gly232 to Pro may affect tetramer formation. The MD average conformations for *Mtu*DAH7PS_WT_, *Mtu*DAH7PS_G232P_ and *Mtu*DAH7PS_WT_^dimer^ were compared with the crystal structure of ligand free *Mtu*DAH7PS_WT_ (PDB 3NV8). Each chain within these MD average structures sampled slightly different average conformations and are all superimposed with chain A of the ligand free crystal structure for comparison, allowing conformational differences particularly around the regions involved in forming the tetramer interfaces to be closely examined (Fig 7C). Helices α2a and α2b in the MD average structure of *Mtu*DAH7PS_WT_ mostly maintained similar conformations to those observed in the ligand free crystal structure (Fig 7C). A range of slightly different conformations were observed around the α2b-β3 loop due to its flexibility, corresponding to the larger RMSD values calculated for this region (residue 235–240) compared to the preceding helical regions (Fig 7B). In contrast to *Mtu*DAH7PS_WT_, both dimeric systems showed much greater changes in the positions of helices α2a and α2b as well as the conformation of the flexible α2b-β3 loop (Fig 7B and 7C). The theoretical dimer *Mtu*DAH7PS_WT_^dimer^ showed similar tilting of the inserted helices to the dimeric variant *Mtu*DAH7PS_G232P_ (Fig 7C), with slightly larger conformational changes in α2a helix and the first half of α2b helix (residues 194–218) in comparison to *Mtu*DAH7PS_G232P_ (Fig 7B). However, for regions that were actively involved in forming the tetramer interface in *Mtu*DAH7PS_WT_, i.e. the second half of helix α2b (residues 223–232) and the α2b-β3 loop (residues 233–237), *Mtu*DAH7PS_G232P_ showed larger deviations in conformation compared to *Mtu*DAH7PS_WT_^dimer^, suggesting that in addition to the changes of positions of the inserted helices caused by purely dimerization, the G232P substitution could contribute to further movements in this region.

In order to investigate the effect on the formation of interfaces caused by the seemingly slight change in conformations in the regions α2a-β3, the tight dimer interface in *Mtu*DAH7PS_G232P_ was analyzed and compared to the dimer interface observed in *Mtu*DAH7PS_WT_. The MD average conformations for both *Mtu*DAH7PS_G232P_ and *Mtu*DAH7PS_WT_ were subjected to interface analysis using PISA (Table 3). Results from PISA showed that the dimer interface in *Mtu*DAH7PS_G232P_ is more extensive and hydrophobic than that of *Mtu*DAH7PS_WT_, as indicated by a larger interface area, a more negative Δ^i^G value and fewer salt bridges across the interface. This increase in dimer interface area was mainly contributed by the conformational changes in α2a-β3 loop in *Mtu*DAH7PS_G232P_. It is possible that the conformational and dynamic changes caused by the G232P substitution favors a more extensive dimer interface, at the expense of disruption to the formation of the tetramer interface. In order to investigate the effect on tetramer formation by the G232P substitution, the potential tetramer interface that would be formed if *Mtu*DAH7PS_G232P_ were tetrameric was also analyzed using PISA. A “pseudo-tetramer” of *Mtu*DAH7PS_G232P_ was generated by aligning two dimers of the *Mtu*DAH7PS_G232P_ MD average conformations to the tetramer crystal structure of *Mtu*DAH7PS_WT_ (PDB 3NV8). The calculations show that this pseudo-tetramer interface in *Mtu*DAH7PS_G232P_ would have reduced interface area and be more polar compared to the tetramer interface in *Mtu*DAH7PS_WT_. The calculated solvation free energy gain upon formation of this pseudo-interface (Δ^i^G) decreased more than 2-fold, in line with it being a less hydrophobic interface in comparison with *Mtu*DAH7PS_WT_. Furthermore, the P-value (a measure of interface specificity) for Δ^i^G of the pseudo-tetramer interface in *Mtu*DAH7PS_G232P_ is greater than 0.5 (0.651 in contrast to P-value of 0.087 in *Mtu*DAH7PS_WT_), which indicates the pseudo-tetramer interface is unlikely to form.

**Table 3:** Interface analysis results for *Mtu*DAH7PS WT and G232P from PISA [29]. Δ^i^G indicates the solvation free energy gain upon formation of the interface. P-value indicates the probability to obtain solvation energy gain lower than the observed value Δ^i^G, if interface atoms were picked randomly from protein surface such as to amount to the observed interface area. An interface with P>0.5 is likely to be an artefact and interfaces with P<0.5 are likely to be interaction specific. N_NH_ and N_SB_ indicate the number of potential hydrogen bonds and salt bridge interactions across the interface, respectively.

	Dimer interface					(Pseudo) tetramer interface				
	Interface area (Å^2^)	Δ^i^G (kcal/mol)	Δ^i^G P-value	N_HB_	N_SB_	Interface area (Å)	Δ^i^G (kcal/mol)	Δ^i^G P-value	N_HB_	N_SB_
***Mtu*DAH7PS**_**WT**_	2054.4	-25.7	0.220	28	15	915.1	-15.9	0.087	4	1
***Mtu*DAH7PS**_**G232P**_	2123.6	-27.8	0.240	28	10	839.7	-6.8	0.651	27	6

## Discussion

The importance of quaternary structure has been noted for both enzyme function and regulation. For example, oligomeric states were found to affect the activity of secondary transport proteins [5] and (β/α)_8_ barrel enzymes [30]; quaternary structures also contribute to the regulation of d-3-phosphoglycerate dehydrogenase (PGDH) from *Mycobacterium tuberculosis* [31] and thymidine kinases [32]. *Mtu*DAH7PS has previously been shown to adopt a highly sophisticated mechanism for its allosteric regulation [18, 19, 21]. This homotetrameric enzyme hosts allosteric binding sites that are selective for each of the three aromatic amino acids (Phe, Tyr and Trp), two of which (the Phe and Trp binding sites) are mainly formed by additional structural elements to the main barrel, and are located at interfaces that are only formed upon oligomerization. It is not inhibited by individual aromatic amino acids, but rather by binary or ternary combinations that include Trp. In this study, we have explored directly the role of quaternary structure in the function and allosteric regulation of *Mtu*DAH7PS.

Dimeric variants of *Mtu*DAH7PS were successfully generated by either disrupting the physical interactions that stabilize the tetramer interface (*Mtu*DAH7PS_F227D_) or by altering the dynamic properties of regions around the tetramer interface via a remote amino acid substitution (*Mtu*DAH7PS_G232P_). It was found, as expected, that allosteric inhibition was significantly impeded upon loss of the tetramer interface. Combinations of aromatic amino acids involving Trp did not show substantial inhibitory effect on the dimeric variants. The binding of Phe was largely unaffected, but, in contrast, the binding of Trp was shown to be significantly attenuated, which was not surprising due to the close proximity of the tetramer interface and the Trp allosteric site in the tetrameric structure of the wild type enzyme.

The dimeric variants also showed impaired catalytic efficiency. Large increases in *K*_m_ values of both PEP and E4P observed in the dimeric variants may suggest that dimerization partially mimics the effect of allosteric inhibition. However, the lack of the homotropic cooperativity behavior with respect to E4P, which was observed for the allosterically inhibited wild type enzyme [18], suggests that the effect of dimerization is not the same as that caused by allosteric ligand binding. Previous study has established that protein dynamics plays an important role in communication between the allosteric and the active sites [24]. In this study, molecular dynamics simulations suggested that the dynamic properties of the enzyme were changed upon dimerization, therefore it is not surprising that the catalytic properties of the active site are also altered in the dimeric variants.

Given the ease of breaking the tetramer interface with just single amino acid substitution as shown in this study, it is reasonable to consider whether a dimer-tetramer equilibrium could be part of the allosteric regulation mechanism of *Mtu*DAH7PS. However, all of the existing experimental evidences suggest that this is not the case. Firstly, combinations of aromatic amino acids that include Trp are required for allosteric regulation of wild type *Mtu*DAH7PS, but the presence of Trp alone is not inhibitory [18]. Trp binds tightly in the tetramer interface in the wild-type enzyme and its binding is significantly attenuated in the dimeric variants. Therefore, the tetrameric species of *Mtu*DAH7PS is both fully active and the inhibited species involved in allosteric regulation. Secondly, chorismate mutase is activated by formation of an enzyme complex with *Mtu*DAH7PS across the tetramer interface, which further stabilizes the tetrameric species of *Mtu*DAH7PS [21, 22]. This species is also fully active and responsive to allosteric regulation of DAH7PS activity. Thirdly, tetrameric wild-type *Mtu*DAH7PS is more active than the dimeric variants created in this study, suggesting that tetramer formation is required for efficient catalysis. Therefore, while it is conceivable that dimeric species of the wild type enzyme may exist under low protein concentrations, the existing experimental evidence suggest that the tetrameric species of *Mtu*DAH7PS is the biologically relevant species, and the dimer-tetramer equilibrium is not required for effective allosteric regulation of *Mtu*DAH7PS.

However, there appears to be an intimate relationship between quaternary structure and allostery in DAH7PS enzymes. The accessory structural elements to the core barrel have been shown to contribute to the formation of interfaces and to be closely linked to allostery. DAH7PS from *Thermotoga maritima* (*Tma*DAH7PS, type 1β) contains an ACT domain (small domain of 60–70 residues with a βαββαβ topology [33, 34]) attached to the main barrel via a flexible linker. Two of the ACT domains come together to form allosteric binding sites for Tyr [25]. DAH7PS from *Neisseria meningitidis* (*Nme*DAH7PS, type 1α) has an N-terminal extension and inserted β-sheets added to the main barrel, and these additional structural elements contribute to the dimer and tetramer interfaces as well as forming the binding site for allosteric ligand Phe [35]. *Mtu*DAH7PS is the only type II DAH7PS so far with a known structure and extensive studies have shown that the N-terminal extension and the inserted helices contribute to the interfaces and are responsible for the highly sophisticated allosteric regulation observed for this enzyme [14, 18, 19, 21, 24]. However, a group of type II DAH7PS enzymes, which are involved in secondary-metabolite biosynthesis, lack the region corresponding to the inserted helices in *Mtu*DAH7PS that are responsible for the formation of the tetramer interface and the Trp binding site [36]. For example, *Pseudomonas aeruginosa* contains two forms of type II DAH7PS (*Pae*DAH7PS), which could be named long and short forms based on the length of their amino acid sequence. The long form *Pae*DAH7PS highly resembles *Mtu*DAH7PS, whereas the short form omits the sequence corresponding to the inserted helices (α2a and α2b) in *Mtu*DAH7PS. Knowing the essential role of these inserted helices in forming the tetramer interface and hosting the allosteric binding site for Trp in *Mtu*DAH7PS, the lack of the inserted helices in the short form *Pae*DAH7PS suggests that it may adopt a different, perhaps dimeric, quaternary structure, and may not be sensitive to inhibition by Trp. Future studies to obtain structural information for other type II DAH7PS may help to further clarify the importance of these additional structural elements and further our understanding of the relationship between quaternary structure and allostery.

## Materials and methods

### Generation of variant *Mtu*DAH7PS, protein expression and purification

The variants of *Mtu*DAH7PS were produced using the QuikChange Lightning Site-Directed Mutagenesis Kit (Stratagene) in accordance with the manufacturer's instructions and using the pPro-Ex-HTa wild-type plasmid as the template. Primers used to produce *Mtu*DAH7PS_F227D_ were Fwd: 5´-CCACAGGCACTCATGTCCCGCAGCCCACGATC-3´ and Rev: 5´- GATCGTGGGCTGCGGGACATGAGTGCCTGTGG-3´, and the primers used to produce *Mtu*DAH7PS_G232P_ were Fwd: 5´-GCGGTTCATGAGTGCCTGTCCGGTGGCCGAC-3´ and Rev: 5´-GTCGGCCACCGGACAGGCAGGCACTCATBAACCGC-3´. The resulting PCR products were transformed into chemically competent One Shot TOP10 (Invitrogen) and electrocompetent BL21 (DE3) pGroESL *E*. *coli* cell lines for plasmid storage and protein expression, respectively. The protein expression and purification of both *Mtu*DAH7PS (including variant *Mtu*DAH7PS) and *Mtu*CM were carried out as previously described by Webby *et al*. and Blackmore *et al*. respectively [14, 18, 21].

### Enzyme assays and kinetic measurements

The *Mtu*DAH7PS activity assays were conducted at 30°C as reported previously reported [14, 18]. Enzyme activity was monitored by following the loss of absorbance at 232 nm due to consumption of PEP. The reaction mixtures contained E4P (25–150 μM), MnSO_4_ (100 μM), and PEP (fixed at 150 μM or varied to determine kinetic parameters) in assay buffer (50 mM BTP, pH 7.5, and 1 mm tris(2-carboxyethyl) phosphine)). PEP and E4P solutions were prepared in assay buffer, and the MnSO_4_ solution was prepared in ultrapure water. The reaction was initiated by the addition of purified *Mtu*DAH7PS. Initial reaction rates were determined by a least squares fit of the initial-rate data.

*K*_*m*_ and *k*_cat_ values for *Mtu*DAH7PS were determined by fitting the data to the appropriate ternary complex kinetic equation using the program Prism 6 (Graphpad). Kinetic data were obtained at three different E4P concentrations (25, 50 and 100 μM for *Mtu*DAH7PS and 50, 75 and 100 μM for the variant enzymes) and at least five different PEP concentrations for each set of assayed E4P concentration (10, 20, 40, 90, 150 and 300 μM for wild-type enzyme; 5, 20, 40, 90, 150 and 300 μM for *Mtu*DAH7PS_G232P_; and 10, 20, 40, 150 and 300 μM for *Mtu*DAH7PS_F227D_).

### Measurement of *Mtu*CM activity

The *Mtu*CM activity assays in the presence of *Mtu*DAH7PS monitored the loss of chorismate by loss of absorbance at 274 nm and were conducted at 30°C as previously reported [21]. Assays to determine the activation of *Mtu*CM were performed with 1:10 molar ratio *Mtu*CM to *Mtu*DAH7PS. Assays were conducted with 150 μM chorismic acid and 60 nM *Mtu*CM. When assayed alone the *Mtu*CM concentration was increased to 90 nM and the results normalised.

### Feedback inhibition studies

Solutions of L-Phe (Sigma), L-Tyr (Sigma), and L-Trp (Sigma) in ultrapure water were added to standard *Mtu*DAH7PS assay reaction mixtures. Inhibition study assays were conducted in the presence of none, one or two aromatic amino acids. *Mtu*DAH7PS steady-state kinetic assay solutions contained PEP (150 μM), E4P (150 μM), MnSO4 (100 μM) and amino acid (0–200 μM) in assay buffer. For assays with two amino acids, the background concentration of the indicated amino acid was held at 100 μM. Reactions were initiated by the addition of *Mtu*DAH7PS.

### Isothermal titration calorimetry

ITC experiments were performed using a VP-ITC microcalorimeter (Microcal, GE Healthcare) at 25°C. Ligands were dissolved into the SEC buffer and pH adjusted to match the pH of the original buffer solution. Prior to each experiment, the protein was buffer exchanged into the SEC buffer used to prepare the ligand solution. The protein concentration was measured by UV absorption at 280 nm. All solutions were filtered and degassed in a vacuum immediately before use. For all experiments, the cell contained a solution of *Mtu*DAH7PS and the syringe contained the ligand solution. Each experiment consisted of 29 injections, one 2 μL injection and 28 subsequent 10 μL injections. The initial data point was deleted to allow for diffusion of the ligand across the needle tip during the initial equilibration period. Heats of dilution were measured independently and subtracted from the titration data before curve fitting of the data using Origin (version 7.0, OriginLab^®^) using the single-site binding model supplied by MicroCal. ITC data is provided in S3 Fig.

### SAXS

SAXS measurements were performed using methods previously described at the SAXS/WAXS beamline located at the Australian Synchrotron [24]. Protein samples at concentrations of 6 mg/mL were added to size-exclusion chromatography column.

Scattering intensity (I) was plotted against *s* [where *s* is the magnitude of the scattering vector, which is related to the scattering angle (2θ) and the wavelength (λ) as follows: *s* = (4π/λ)sinθ]. All samples were assessed for non-linearity in the increase of intensity at low s to check for aggregation or concentration dependent scattering. 1D profiles were background subtracted and Guinier fits were made using PRIMUS and found to be linear for *s*·*R*_g_<1.3 (*R*_g_ is the radius of gyration). An indirect Fourier transform was performed by GNOM to provide a pair wise distribution function (P(r)) which determined both the relative probability of scattering centres being separated by a given distance and the maximum dimension of the scattering particle (*D*_max_). Theoretical scattering curves were generated from atomic coordinates (PDB 3NV8) and compared with experimental scattering curves using CRYSOL [37]. SAXS parameters from the analyses are presented in Table 4.

**Table 4:** SAXS parameters calculated from analysis of the scattering profiles of *Mtu*DAH7PS variants using PRIMUS, GNOM, MoW, and CRYSOL. Values were obtained by analysis of one set of SAXS data.

	*Mtu*DAH7PS_WT_	*Mtu*DAH7PS_G232P_	*Mtu*DAH7PS_F227D_
Data points	379	207	297
q-range	0.014–0.265	0.019–0.269	0.014–0.269
*Guinier analysis*			
*R*_g_ (Å)	41.6 ± 0.3	29.8 ± 0.1	29.8 ± 0.1
*I* (0) (cm^-1^)	0.168 ± 0.001	0.074 ± 0.001	0.066 ± 0.001
*Pair distribution analysis*			
Real Space *R*_g_ (Å)	40.9 ± 0.2	29.6 ± 0.1	28.83 ± 0.07
*D*_max_ (Å)	127	98	96
*V*_p_, (Da)	282700	144500	150518
*Molecular weight estimates from SAXS MoW analysis*			
M_w_ from *V*_p_ (Da)	199 100	89800	91800
Nō of Subunits	4	2	2
*CRYSOL analysis with Tetrameric 3NV8*			
*R*_gE_ (Å)	42.4 ± 0.2	41.6 ± 0.2	29.8 ± 0.2
*R*_gT_ (Å)	40.6	29.6 ± 0.2	39.8
χ	0.996	40.0	51.4
*CRYSOL analysis with dimer of* Mtu*DAH7PS 3NV8 in asymmetric unit*			
*R*_gE_ (Å)	41.7 ± 0.2	29.6 ± 0.2	29.9 ± 0.2
*R*_gT_ (Å)	30.7	29.1	29.3
χ	13.3	0.986	2.36

### Analytical ultracentrifugation (AUC)

Sedimentation velocity experiments were performed in a Beckman Coulter XL-1 analytical ultracentrifuge equipped with UV/Vis scanning optics at 20°C. All protein samples were prepared by dialysis overnight into the buffer solution (50 mM Tris buffer with 150 mM NaCl and 200 μM MnSO_4_ at pH 7.5). Reference buffer solution (400 μL) and sample solution (380 μL) were loaded into 12 mm double-sector cells with quartz windows, and mounted in an An-50 Ti 8-hole rotor. Protein samples at concentrations of 0.09, 0.6 and 0.9 mg/mL (corresponding to 1.8, 12 and 18 μM respectively) were centrifuged at 50,000 rpm and the absorbance data was collected at 280 or 290 nm without averaging. For AUC experiments in the presence of 100 μM Phe and 100 μM Trp, both the protein sample and the buffer reference were spiked with the amino acid just prior to analysis. AUC data was fitted to a continuous distribution *c*(*s*) model using SEDFIT [38, 39]. The partial specific volume (v) of the protein samples, butter density (1.005 gmL^-1^) and buffer viscosity (1.021 cp) were calculated using the program SEDTERP [40]. Sedimentation velocity data for *Mtu*DAH7PS_G232P_ in the absence and presence of 100 μM Trp and 100 μM Phe is provided in S4 Fig.

### Molecular dynamics simulations

The MD simulations were carried out with NAMD running on the BlueFern supercomputer at the University of Canterbury [41]. For MD simulation of *Mtu*DAH7PS_WT_^dimer^, the dimeric molecule found in the asymmetric unit of ligand free wild type *Mtu*DAH7PS (PDB 3NV8) was used as starting structure. The crystal structure of ligand free *Mtu*DAH7PS (PDB 3NV8), was also used to prepare input structures for MD simulations of the wild-type *Mtu*DAH7PS tetramer and the dimeric variant *Mtu*DAH7PS_G232P_. For the wild-type tetramer, the quaternary structure was generated from the dimer in the asymmetric unit based on the 2-fold crystallographic symmetry operation. For *Mtu*DAH7PS_G232P_, *in silico* amino acid substitutions were conducted on corresponding residues based on the dimer present in the unit cell of the wild type crystal structure. Each system was solvated with explicit TIP3 water molecules in a box in VMD [42], Na^+^ and Cl^-^ ions were added to balance the net charge of the water box. The ions were added with a minimum distance of 5 Å to the enzyme molecule and to each other. All MD simulations conducted in this study and in previous studies of *Mtu*DAH7PS enzymes [24] contain Mn^2+^ ions at the metal binding sites in the active sites. When Mn^2+^ ions were not present in the initial crystal structures, they were included by manually adding the ions to the corresponding metal binding sites. MD simulations were conducted with the CHARMM22 all-hydrogen parameter file for proteins at a constant temperature and pressure (310 K, 1 atm) [43]. The cutoff distance for van der Waals interactions was set to 12 Å. In each simulation, the system was first minimized for 5000 steps followed by dynamics simulation conducted with 2 fs time steps. The MD simulations were conducted for 464.1 ns, 429.9 ns and 540.9 ns for *Mtu*DAH7PS_G232P_, *Mtu*DAH7PS_WT_ and *Mtu*DAH7PS_WT_^dimer^ systems respectively.

## Supporting information

S1 Fig Protein backbone RMSD values during MD simulations. RMSD values of protein backbone atoms in *Mtu*DAH7PS_WT_ (green), *Mtu*DAH7PS_WT_^dimer^ (red) and *Mtu*DAH7PS_G232P_ (blue) are plotted against simulation time (ns).
(TIFF)

S2 Fig Comparison between calculated RMSF values and temperature factors obtained from crystal structure. (A) Calculated chain-average RMSF values for *Mtu*DAH7PS_WT_ during MD simulation. (B) Square root values of the chain-average temperature factor obtained from ligand free crystal structure (PDB 3NV8) of *Mtu*DAH7PS_WT_.
(TIFF)

S3 Fig ITC data. ITC data obtained for (A) 30 μM *Mtu*DAH7PS^G232P^ with 3 mM Trp titrant; (B) 28 μM of *Mtu*DAH7PS^G232P^ with 900 μM Phe titrant; (C) 24 μM *Mtu*DAH7PS^G232P^ and a background of 50 μM Phe present in the cell with a 5 mM Trp titrant; (D) 32 μM *Mtu*DAH7PS^F227D^ with 5 mM Trp titrant; (E) 32 μM of *Mtu*DAH7PS^F227D^ with 600 μM Phe titrant and (F) 28 μM *Mtu*DAH7PS^F227D^ and a background of 50 μM Phe present in the cell with a 5 mM Trp titrant.
(TIFF)

S4 Fig AUC data. Sedimentation velocity data for *Mtu*DAH7PS_G232P_ in the absence and presence of 100 μM Trp and 100 μM Phe. Data collected at 20°C and 50,000 rpm. (A), (B) and (C) are the data from *Mtu*DAH7PS_G232P_ collected at 0.09, 0.6 and 0.9 mg.mL^-1^ and (D), (E) and (F) are the data from *Mtu*DAH7PS_G232P_ collected in the presence of 100 μM Trp and 100 μM Phe. at 0.09, 0.6 and 0.9 mg.mL^-1^. Each panel shows the sedimentation velocity data (data points), the size-distribution best fit (solid lines) and residuals for the data fits (top panels).
(TIFF)
