# Supplementary figures and images for: Quaternary structure is an essential component that contributes to the sophisticated allosteric regulation mechanism in a key enzyme from Mycobacterium tuberculosis
Source: PLoS One. 2017 Jun 30;12(6):e0180052. doi: 10.1371/journal.pone.0180052 (PMC5493349; doi:10.1371/journal.pone.0180052)

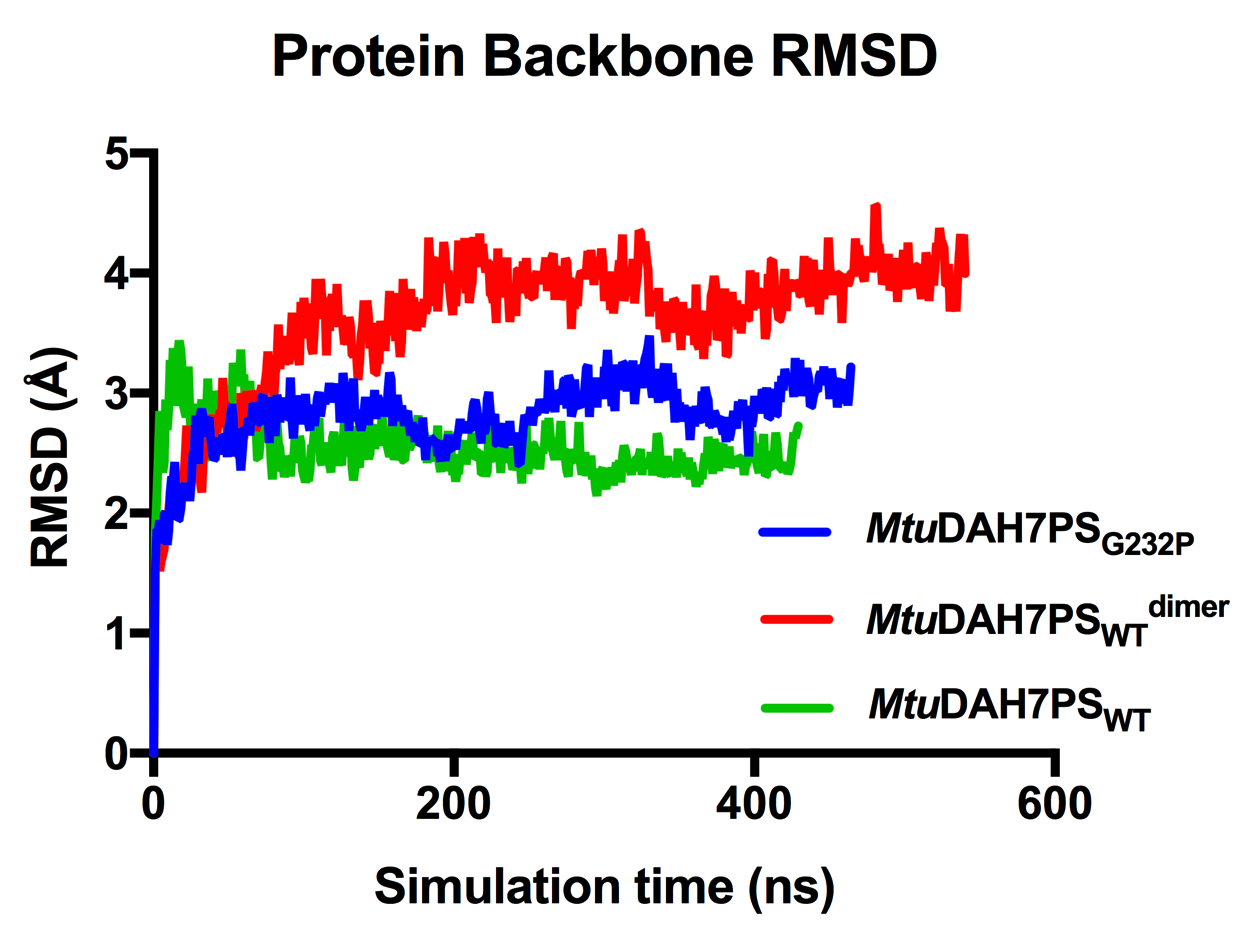

Supplement: S1 Fig — RMSD values of protein backbone atoms in MtuDAH7PSWT (green), MtuDAH7PSWTdimer (red) and MtuDAH7PSG232P (blue) are plotted against simulation time (ns). (TIFF) [file pone.0180052.s001.tiff]

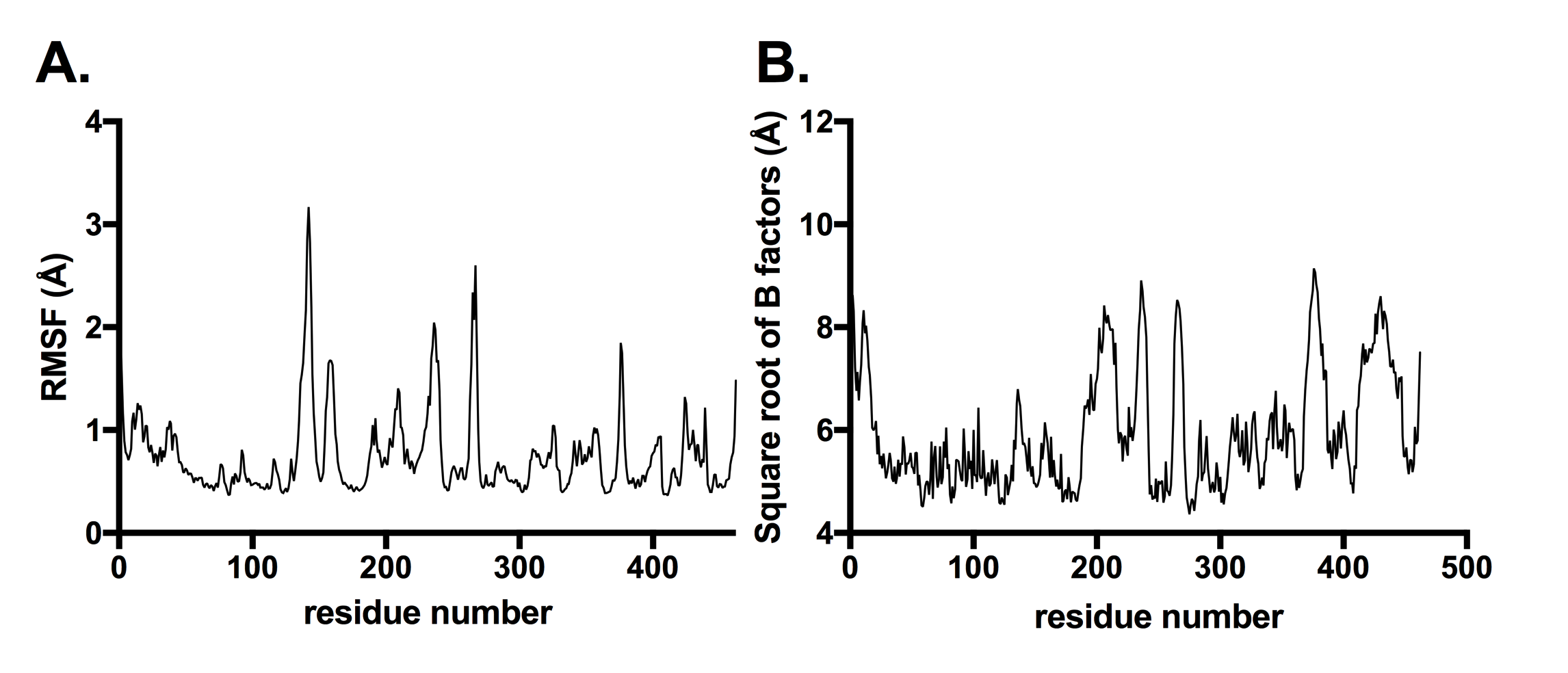

Supplement: S2 Fig — (A) Calculated chain-average RMSF values for MtuDAH7PSWT during MD simulation. (B) Square root values of the chain-average temperature factor obtained from ligand free crystal structure (PDB 3NV8) of MtuDAH7PSWT. (TIFF) [file pone.0180052.s002.tiff]

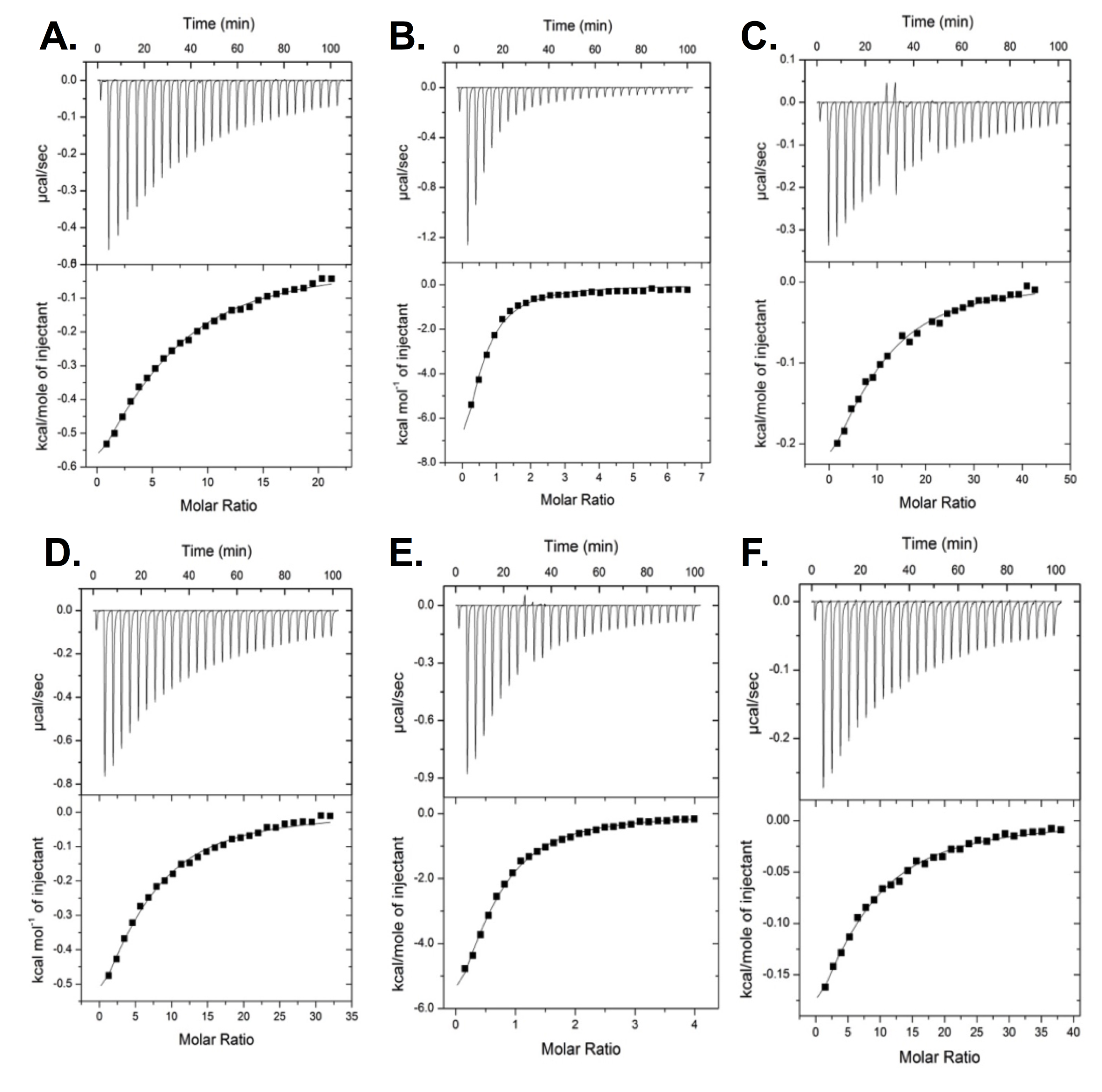

Supplement: S3 Fig — ITC data obtained for (A) 30 μM MtuDAH7PSG232P with 3 mM Trp titrant; (B) 28 μM of MtuDAH7PSG232P with 900 μM Phe titrant; (C) 24 μM MtuDAH7PSG232P and a background of 50 μM Phe present in the cell with a 5 mM Trp titrant; (D) 32 μM MtuDAH7PSF227D with 5 mM Trp titrant; (E) 32 μM of MtuDAH7PSF227D with 600 μM Phe titrant and (F) 28 μM MtuDAH7PSF227D and a background of 50 μM Phe present in the cell with a 5 mM Trp titrant. (TIFF) [file pone.0180052.s003.tiff]

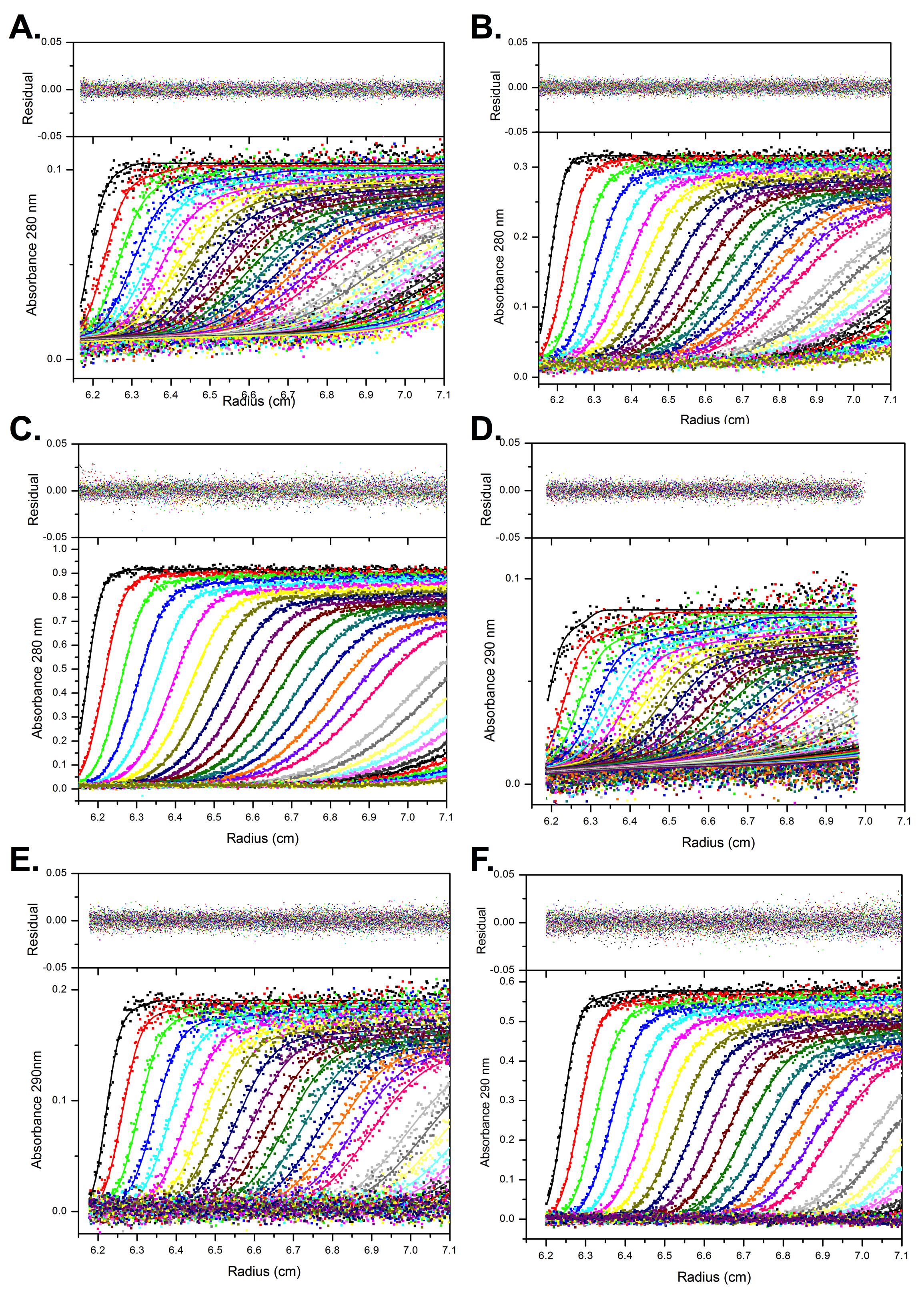

Supplement: S4 Fig — Sedimentation velocity data for MtuDAH7PSG232P in the absence and presence of 100 μM Trp and 100 μM Phe. Data collected at 20°C and 50,000 rpm. (A), (B) and (C) are the data from MtuDAH7PSG232P collected at 0.09, 0.6 and 0.9 mg.mL-1 and (D), (E) and (F) are the data from MtuDAH7PSG232P collected in the presence of 100 μM Trp and 100 μM Phe. at 0.09, 0.6 and 0.9 mg.mL-1. Each panel shows the sedimentation velocity data (data points), the size-distribution best fit (solid lines) and residuals for the data fits (top panels). (TIFF) [file pone.0180052.s004.tiff]
